# Triosephosphate Isomerase Gene Characterization and Potential Zoonotic Transmission of *Giardia duodenalis*

**DOI:** 10.3201/eid0911.030084

**Published:** 2003-11

**Authors:** Irshad M. Sulaiman, Ronald Fayer, Caryn Bern, Robert H. Gilman, James M. Trout, Peter M. Schantz, Pradeep Das, Altaf A. Lal, Lihua Xiao

**Affiliations:** * Centers for Disease Control and Prevention, Atlanta, Georgia, USA; † U.S. Department of Agriculture, Beltsville, Maryland, USA; ‡ Johns Hopkins School of Public Health, Baltimore, Maryland, USA; § National Institute of Cholera and Enteric Diseases, Calcutta, West Bengal, India

## Abstract

To address the source of infection in humans and public health importance of *Giardia duodenalis* parasites from animals, nucleotide sequences of the triosephosphate isomerase (TPI) gene were generated for 37 human isolates, 15 dog isolates, 8 muskrat isolates, 7 isolates each from cattle and beavers, and 1 isolate each from a rat and a rabbit. Distinct genotypes were found in humans, cattle, beavers, dogs, muskrats, and rats. TPI and small subunit ribosomal RNA (SSU rRNA) gene sequences of *G. microti* from muskrats were also generated and analyzed. Phylogenetic analysis on the TPI sequences confirmed the formation of distinct groups. Nevertheless, a major group (assemblage B) contained most of the human and muskrat isolates, all beaver isolates, and the rabbit isolate. These data confirm that *G. duodenalis* from certain animals can potentially infect humans and should be useful in the detection, differentiation, and taxonomy of *Giardia* spp.

Giardiasis is a common cause of diarrheal disease in almost all vertebrates, including humans. In industrialized countries, it is referred to as a reemerging infectious disease because of its increasingly recognized role in outbreaks of diarrheal disease in daycare centers and in water- and foodborne outbreaks. *Giardia* is also one of the most frequently observed parasites infecting dairy cattle and domestic dogs. In developing countries in Asia, Africa, and Latin America, approximately 200 million people have symptomatic giardiasis (*1*).

The taxonomy of *Giardia* at the species level is complicated and unresolved because of limited morphologic differences. Based on morphology, six species of this genus are considered valid: *Giardia duodenlalis* (syn. *G. lamblia* or *G. intestinalis*) in a wide range of mammals, including humans, livestock, and companion animals; *G. agilis* in amphibians; *G. muris* in rodents; *G. ardeae* and *G. psittaci* in birds; and *G. microti* in muskrats and voles (*2*–*6*). However, on the basis of host origins, 41 *Giardia* species have been named (*7*,*8*).

Molecular tools have been used recently to characterize the epidemiology of human giardiasis. Although isolates of *G. duodenalis* from humans and various animals are morphologically similar, distinct host-adapted genotypes have been demonstrated within *G. duodenalis* (*1*,*9*–*12*). Two major groups of *G. duodenalis* have been recognized as infecting humans worldwide, but there are some differences in naming of these groups, as evidenced by the following categorizations: Polish and Belgian genotypes (*9*); groups 1, 2, and 3 (*10*,*13*); and assemblages A and B (*11*). So far, no general consensus has been reached concerning the nomenclature of these genotypes, but the term assemblages has been more widely used. The finding of host-adapted *Giardia* genotypes is of public health importance, considering the controversy regarding the zoonotic potential of *Giardia* (*1*,*14*).

We describe the development of a two-step nested polymerase chain reaction (PCR) protocol to amplify the triosephosphate isomerase (TPI) gene of *G. duodenalis* and *G. microti* and nucleotide sequence characterization of amplified TPI fragment. The TPI gene was chosen because of the high genetic heterogeneity displayed by *Giardia* spp. at this locus (*12*,*15*). Results of the study have validated previous observations on the genetic diversity of *Giardia* parasites on the basis of characterization of the glutamate dehydrogenase (GDH), small subunit ribosomal RNA (SSU rRNA), and TPI genes (*12*,*15*–*17*). These data also suggest that some animal isolates of *G. duodenalis* are of zoonotic potential. These data should be useful in developing alternative molecular tools to differentiate *Giardia* parasites at species and genotype levels and in investigating giardiasis outbreaks or endemic diseases.

## Materials and Methods

### *G. duodenalis* Isolates and DNA Extraction

Fecal samples containing *G. duodenalis* cysts were obtained from infected humans, cattle, companion animals (dogs and a rabbit), aquatic wildlife (beavers and muskrats), and one rat. Human samples were mostly from sporadic cases, with the exception of two isolates (4599, 4600) from a foodborne outbreak. Fecal samples with *G. microti* were obtained from infected muskrats. *Giardia* infection was diagnosed by microscopy of wet mounts or immunofluorescence-stained materials. Samples were stored at 4°C in 2.5% (w/v) potassium dichromate solution or frozen at –20°C and used in DNA extraction without cyst isolation (Table 1). For DNA extraction, 200 μL of the fecal suspension from each sample was aliquoted and washed three times with distilled water. The material was treated initially with 66.7 μL of 1 M KOH and 18.6 μL of 1 M dithiothreitol (DTT) followed by neutralization with 8.6 μL of 25% (v/v) hydrochloric acid. The DNA lysate was then extracted once with phenol:chloroform:isoamyl alcohol (25:24:1) solution, and purified by using the QIAamp DNA Stool Kit (QIAGEN Inc, Valencia, CA). Because the extracted DNA contained nucleic acids from both *Giardia* cysts and fecal materials, DNA concentration was not determined for all samples.

**Table 1:** *Giardia* isolates with genotype identity

Isolate	Location	Y	Host	Genotype/species	G+C content (%)
2875	Lima, Peru	2001	Human	Assemblage A	56.3
2891	Lima, Peru	2001	Human	Assemblage A	56.2
2893	Lima, Peru	2001	Human	Assemblage A	56.3
2905	Lima, Peru	2001	Human	Assemblage A	56.1
2907	Lima, Peru	2001	Human	Assemblage A	56.3
2922	Lima, Peru	2001	Human	Assemblage A	56.1
341	Hyderabad, India	1998	Human	Assemblage B	52.1
2578	Calcutta, India	2000	Human	Assemblage B	51.7
2579	Calcutta, India	2000	Human	Assemblage B	51.7
2580	Calcutta, India	2000	Human	Assemblage B	51.6
2582	Calcutta, India	2000	Human	Assemblage B	51.2
2583	Calcutta, India	2000	Human	Assemblage B	51.4
2586	Calcutta, India	2000	Human	Assemblage B	51.6
2587	Calcutta, India	2000	Human	Assemblage B	51.9
2589	Calcutta, India	2000	Human	Assemblage B	51.7
2590	Calcutta, India	2000	Human	Assemblage B	51.7
2506	Lima, Peru	2000	Human	Assemblage B	51.7
2536	Lima, Peru	2000	Human	Assemblage B	51.7
2877	Lima, Peru	2001	Human	Assemblage B	51.3
2879	Lima, Peru	2001	Human	Assemblage B	51.9
2887	Lima, Peru	2001	Human	Assemblage B	51.5
2890	Lima, Peru	2001	Human	Assemblage B	51.9
2895	Lima, Peru	2001	Human	Assemblage B	51.9
2900	Lima, Peru	2001	Human	Assemblage B	51.4
2901	Lima, Peru	2001	Human	Assemblage B	51.1
2902	Lima, Peru	2001	Human	Assemblage B	52.1
2913	Lima, Peru	2001	Human	Assemblage B	51.5
2915	Lima, Peru	2001	Human	Assemblage B	51.5
2917	Lima, Peru	2001	Human	Assemblage B	51.5
2920	Lima, Peru	2001	Human	Assemblage B	51.6
2924	Lima, Peru	2001	Human	Assemblage B	51.9
2926	Lima, Peru	2001	Human	Assemblage B	51.9
2930	Lima, Peru	2001	Human	Assemblage B	51.1
2932	Lima, Peru	2001	Human	Assemblage B	51.5
2935	Lima, Peru	2001	Human	Assemblage B	51.6
4599	San Francisco, CA	2001	Human	Assemblage B	51.9
4600	San Francisco, CA	2001	Human	Assemblage B	51.8
1758	Changchun, China	2000	Rabbit	Assemblage B	50.7
1653	Preston, MD	2000	Beaver	Assemblage B	50.7
1654	Preston, MD	2000	Beaver	Assemblage B	50.7
1655	Preston, MD	2000	Beaver	Assemblage B	50.9
3495	Preston, MD	2001	Beaver	Assemblage B	50.5
3500	Preston, MD	2001	Beaver	Assemblage B	51.1
3518	Preston, MD	2001	Beaver	Assemblage B	51.1
3599	Preston, MD	2001	Beaver	Assemblage B	50.7
3469	Preston, MD	2001	Muskrat	Assemblage B	50.9
3470	Preston, MD	2001	Muskrat	Assemblage B	50.9
3565	Preston, MD	2001	Muskrat	Assemblage B	50.9
3569	Preston, MD	2001	Muskrat	Assemblage B	50.7
3577	Preston, MD	2001	Muskrat	Assemblage B	50.1
867	Atlanta, GA	1999	Dog	Assemblage C	56.1
868	Atlanta, GA	1999	Dog	Assemblage C	56.4
894	Atlanta, GA	1999	Dog	Assemblage C	56.4
895	Atlanta, GA	1999	Dog	Assemblage C	55.5
898	Atlanta, GA	1999	Dog	Assemblage C	56.4
2643	Atlanta, GA	1999	Dog	Assemblage C	56.2
2645	Atlanta, GA	1999	Dog	Assemblage C	55.8
2661	Atlanta, GA	1999	Dog	Assemblage C	55.9
2664	Atlanta, GA	1999	Dog	Assemblage C	56.4
2665	Atlanta, GA	1999	Dog	Assemblage C	55.8
2668	Atlanta, GA	1999	Dog	Assemblage C	56.2
2669	Atlanta, GA	1999	Dog	Assemblage C	56.2
2670	Atlanta, GA	1999	Dog	Assemblage C	56.1
2674	Atlanta, GA	1999	Dog	Assemblage C	55.8
2679	Atlanta, GA	1999	Dog	Assemblage C	56.6
15	Columbus, OH	1997	Cattle	Assemblage E	50.4
109	Columbus, OH	1997	Cattle	Assemblage E	50.6
110	Columbus, OH	1997	Cattle	Assemblage E	50.5
111	Columbus, OH	1997	Cattle	Assemblage E	50.6
112	Columbus, OH	1997	Cattle	Assemblage E	50.9
138	Columbus, OH	1997	Cattle	Assemblage E	50.8
5009	Beltsville, MD	2001	Cattle	Assemblage E	50.5
2135	St Louis, MO	2000	Rat	Assemblage undefined	58.6
3460	Preston, MD	2001	Muskrat	*G. microti*	57.9
3463	Preston, MD	2001	Muskrat	*G. microti*	58.2
3464	Preston, MD	2001	Muskrat	*G. microti*	50.9

### PCR Amplification of the TPI Gene

To amplify the TPI fragment from various *Giardia* isolates, a nested PCR protocol was developed that used primers complementary to the conserved published TPI nucleotide sequences of various *Giardia* parasites downloaded from GenBank: *G. duodenalis* (U57897, AF06957 to AF069563, L02116, L02120), *G. muris* (AF069565), and *G. ardeae* (AF069564). For the primary PCR, a PCR product of 605 bp was amplified by using primers AL3543 [5′-AAATIATGCCTGCTCGTCG-3′] and AL3546 [5′-CAAACCTTITCCGCAAACC-3′]. The PCR reaction comprised 0.25–2.0 μL of DNA, 200 μM each of deoxynucleoside triphosphate (dNTP), 1X PCR buffer (Perkin Elmer, Wellesley, MA), 3.0 mM MgCl_2_, 5.0 U of *Taq* polymerase (GIBCO BRL, Frederick, MD), and 200 nM of each primer in a total of 100-μL reaction. The reactions were performed for 35 cycles (94°C for 45 s, 50°C for 45 s, and 72°C for 60 s) in a Perkin-Elmer GeneAmp PCR 9700 thermocycler, with an initial hot start (94°C for 5 min) and a final extension (72°C for 10 min). For the secondary PCR, a fragment of 530 bp was amplified by using 2.5 μL of primary PCR reaction and primers AL3544 [5′-CCCTTCATCGGIGGTAACTT-3′] and AL3545 [5′-GTGGCCACCACICCCGTGCC-3′]. The conditions for the secondary PCR were identical to the primary PCR. The PCR products were analyzed by agarose gel electrophoresis and visualized after ethidium bromide staining.

### PCR Amplification of the SSU rRNA Gene

A nested PCR protocol was also developed to amplify the SSU rRNA fragment from *Giardia* isolates, using primers complementary to the conserved published SSU rRNA nucleotide sequences from various *Giardia* parasites downloaded from GenBank: *G. duodenalis* (AJ278959, AJ293295 to AJ293299, AJ293300, AJ293301, L29129, M54878, U09491, U09492, X52949), *G. microti* (AF006676, AF006677)*, G. muris* (X65063), and *G. ardeae* (Z17210). For the primary PCR, a PCR product of 300 bp was amplified by using primers AL4303 [5′-ATCCGGTCGATCCTGCCG-3′] and reverse AL4305 [5′-AGGATCAGGGTTCGACT-3′]. The PCR reaction was performed by using the GC-RICH PCR System kit, which consisted of GC-RICH Enzyme mix (Taq polymerase in combination with a proofreading polymerase), GC-RICH-PCR reaction buffer (includes a final 1.5 mM MgCl_2_ and dimethyl sulfoxide [DMSO]), and GC-RICH resolution solution (Roche Diagnostics, Indianapolis, IN) with 0.25–2.0 μL of DNA, 200 μM each of dNTP, and 200 nM of each primer in a total of 50-μL reaction. For the secondary PCR, a fragment of 255 bp was amplified with the GC-RICH PCR System kit (Roche) with 2.5 μL of primary PCR reaction, and 200 nM of primers AL4304 [5′-CGGTCGATCCTGCCGGA-3′] and AL4306 [5′-GGCGGAGGATCAGGGT-3′]. The cycling conditions for both SSU RNA primary and secondary PCR were identical to those used to amplify the TPI gene.

### DNA Sequencing and Phylogenetic Analysis

The secondary PCR products were purified by using Microcon PCR Centrifugal Filter Devices (Millipore Corp., Bedford, MA) and sequenced on an ABI 3100 automated sequencer by using the Big Dye Terminator Cycle Sequencing Ready Reaction Kit (Perkin-Elmer). Sequence accuracy was confirmed by two-directional sequencing of two separate PCR products. Multiple alignment of the nucleotide sequences was performed by using Wisconsin Package Version 9.0 program (*18*). A phylogenetic analysis was performed on the aligned sequences to assess the extent of genetic diversity within *G. duodenalis* parasites as well as their evolutionary relationships with other *Giardia* species. In this analysis, published TPI nucleotide sequences representing *G. duodenalis* (from humans, cattle, cat, dog, muskrat, pig, and rat), *G. muris*, and *G. ardeae* were aligned with TPI sequences of *Giardia* parasites obtained in this study.

A neighbor-joining tree (*19*) was constructed on the basis of the evolutionary distances calculated by the Kimura-2-parameter model using the TreeconW program (*20*). A sequence of *G. ardeae* (GenBank accession no. AF069564) was used as the outgroup since the construction of an unrooted tree showed it to be the most divergent member under analysis. The reliability of these trees was assessed by using the bootstrap method (*21*) with 1,000 pseudoreplicates; values >70% were reported (*22*). Nucleotide sequences of the TPI gene of *G. duodenalis* from humans, cattle, dogs, muskrat, rat, and rabbit, representing different genotypes, were deposited in GenBank under accession numbers AY228628 to AY228649.

A similar phylogenetic analysis was carried out on the nucleotide sequences of the SSU rRNA gene from *G. microti* in muskrats. SSU rRNA nucleotide sequences were deposited in GenBank under accession numbers AY228332 and AY228333.

## Results

PCR products of the expected size (approximately 500 bp) were generated from all 76 isolates. All were sequenced, and all of the nucleotide sequences obtained belonged to the TPI sequences of *Giardia* based on BLAST search of the GenBank database. The sources of these isolates were humans (37 isolates), dogs (15 isolates), muskrats (8 isolates), cattle (7 isolates), beavers (7 isolates), rabbit (1 isolate) and rat (1 isolate). The TPI gene of *Giardia* parasites was rich in GC content, ranging from 50.1% to 58.2% (Table 1). Isolates within each genotype, however, had very similar GC contents in the TPI gene.

The extent of genetic diversity in the genus *Giardia* was assessed by multiple alignments of the TPI nucleotide sequences followed by estimates of genetic distances (Table 2). The analysis showed distinct sequences for the human, cattle, beaver, dog, muskrat, and rat isolates; most animals had one genotype, and humans and muskrats had two genotypes. The genetic polymorphism in *Giardia* parasites was evident along the entire TPI gene both at the interspecies (*Giardia* spp.) and intraspecies (*G. duodenalis*) levels.

**Table 2:** Evolutionary genetic distances between different *Giardia* species and *Giardia duodenalis* assemblages^a^

	*G. ardeae*	*G. muris*	Undefined cat	Assemblage A	Assemblage E	Undefined rat	Assemblage C	Assemblage B	*G. microti*
*G. ardeae*	0.00	19.25	43.08	45.31	52.46	46.85	46.49	50.41	32.28
*G. muris*		0.00	46.53	47.48	47.40	47.38	47.14	47.40	44.26
undefined cat			0.00	10.53	12.82	19.16	22.40	24.34	32.23
Assemblage A				0.00	12.85	17.31	19.14	22.31	30.73
Assemblage E					0.00	23.07	22.35	25.54	36.88
undefined Rat						0.00	16.57	20.49	32.03
Assemblage C							0.00	21.69	27.72
Assemblage B								0.00	34.77
*G. microti*									0.00

**Figure 1:**
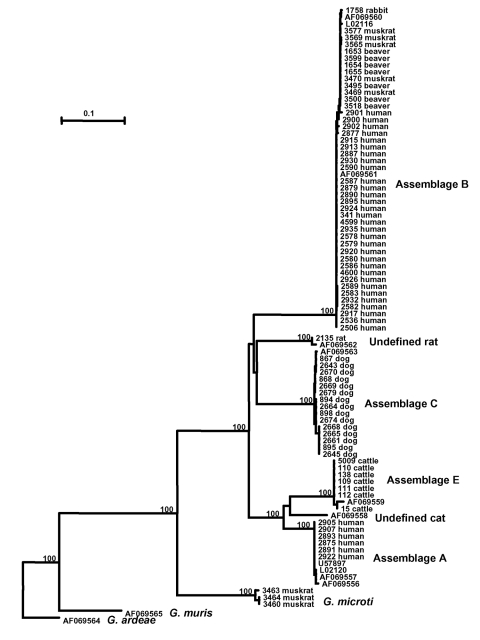
Phylogenetic relationships of *Giardia* parasites inferred by the neighbor-joining analysis of the triosephosphate isomerase (TPI) nucleotide sequences.

To understand the genetic structure of *Giardia* parasites, a neighbor-joining tree was constructed in a phylogenetic analysis of aligned TPI gene sequences of various *Giardia* species and *G. duodenalis* genotypes; we used the nucleotide sequence of *G. ardeae* (AF069564) as an outgroup to root the tree (Figure 1). The phylogenetic analysis showed four distinct clusters for the genus *Giardia*. The first cluster consisted of all isolates of *G. duodenalis* from various sources (humans, cattle, cats, dogs, beavers, muskrats, pigs, and rats). The second cluster consisted of some of the isolates from muskrats. The third and fourth cluster was each represented by a single published sequence of *G. muris* (AF069565) and *G. ardeae* (AF069564).

Several large groups were in the *G. duodenalis* cluster. A major group (assemblage B) was formed with most of the human and muskrat isolates, all the isolates from beavers, and the rabbit isolate (Figure 1). The remaining human isolates aligned with other previously reported human TPI sequences and formed a distinct cluster (assemblage A). Distinct clusters were also evident for the isolates from dogs (assemblage C) and rats (undefined). The cattle sequences, together with the published pig TPI sequence, also formed a distinct cluster (assemblage E or hoofed livestock genotype). Phylogenetic analysis indicated that assemblages B and C and the rat genotype were related to each other and that assemblages A and E and the cat genotype were related to each other. The formation of all major groups was supported by bootstrap analysis with full statistical reliability (Figure 1).

**Figure 2:**
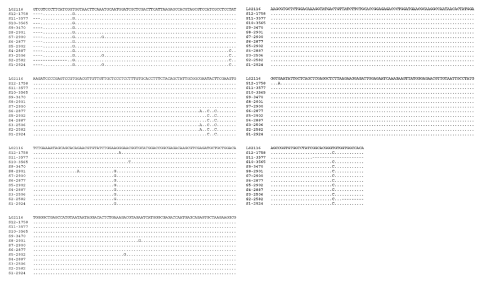
Variation in the triosephosphate isomerase (TPI) nucleotide sequences of *G. duodenalis* isolates belonging to the assemblage B. Twelve distinct subtypes of *G. duodenalis* based on the these sequences were evident within assemblage B. The isolates representing these subtypes (S1–S12) as follows: S1 (341, 2578, 2579, 2580, 2586, 2587, 2879, 2890, 2895, 2920, 2924, 2926, 2935, 4599, 4600); S2 (2582, 2583, 2589, 2932); S3 (2506, 2536, 2917); S4 (2590, 2887, 2913, 2915, 2930); S5 (2902); S6 (2877); S7 (2900); S8 (2901); S9 (1653, 1654, 1655, 3469, 3470, 3495, 3500, 3518, 3599); S10 (3565, 3569); S11 (3577); and S12 (1758). Dots denote sequence identity to GenBank accession no. L02116; dashes denote sequence information not obtained.

Intragenotypic variations were evident within assemblages A, B, C, and E (Table 3). A very high degree of polymorphism was noticed within the isolates from humans. The human isolates grouped in assemblage B had five SNPs (single nucleotide polymorphisms): A or G at position 39, C or T at position 91, G or A at position 162, C or T at position 165, and C or T at position 168 (position numbers according to the GenBank accession no. L02116). Within assemblage B, 12 subtypes of *G. duodenalis* were noticed; 11 of these had not been reported before (Figure 2). No genetic polymorphism was evident in the TPI sequences of the beaver isolates characterized so far, which were identical to those from most muskrats belonging to the major assemblage B group. However, two muskrat isolates (3565, 3569) in assemblage B had one SNP at position 216 (C to T). Six SNPs (A or G at position 51, T or C at position 77, T or G at position 150, C or T at position 330, T or C at position 383, and C or A at position 393) were evident within the dog isolates (assemblage C). Multiple alignments of sequences from hoofed livestock showed two distinct subtypes in cattle with four SNPs (T or C at position 72, G or T at position 78, T or C at position 93, and G or A at position 109). The sequence from the rat matched with the TPI sequence from another suckling mouse (GenBank accession no. AF069562) with one SNP (G to A) at position 54. No genetic variation was observed in the human TPI sequences of assemblage A generated in this study, even though three sequences from GenBank (AF069556, L02120, and U57897) had three SNPs.

**Table 3:** Number of genotypes present in each assemblage

Assemblage	No. of isolates studied	No. of subtypes
A	6	1
B	44	12
C	15	4
E	7	2
Undefined rat	1	1
*Giardia microti*	3	2

**Figure 3:**
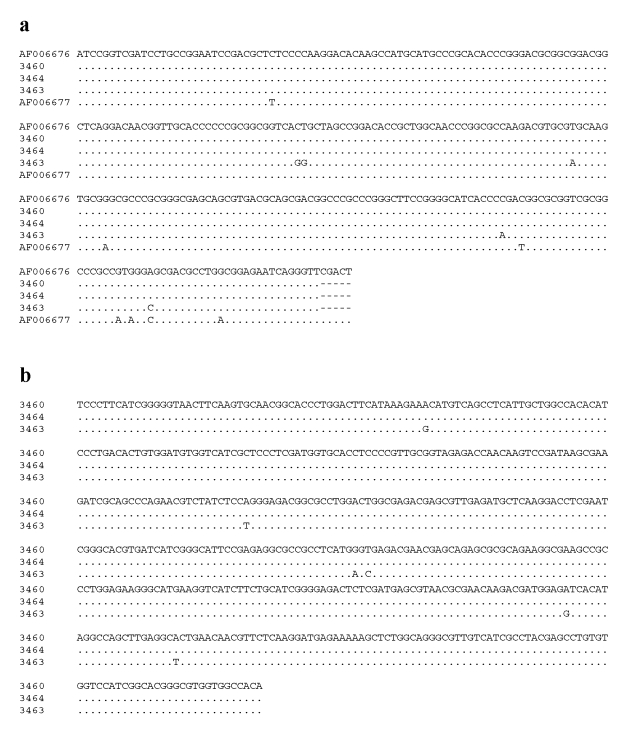
Genetic variation in the nucleotide sequences of *Giardia* *microti* parasites in the small subunit ribosomal RNA (SSU rRNA) (a) and triosephosphate isomerase (TPI) (b) genes.

Since TPI nucleotide sequences of three isolates from muskrats were very different from known *G. duodenalis* isolates or with *C. muris* or *C. ardeae* and since they formed a distinct cluster, these isolates were characterized at the SSU rRNA locus. The *Giardia* SSU rRNA sequences obtained were aligned with the published sequences. Analysis showed that these isolates were *G. microti*. Of the three muskrat SSU rRNA sequences from this study, two (isolates 3460 and 3464) were identical to a published SSU rRNA sequence (AF006676) from muskrats (*6*). The third sequence (isolate 3463) was unique and had three SNPs compared with the other two muskrat isolates and AF006676. Isolate 3463 was still considered to be a sequence of *G. microti* because another published *G. microti* sequence (AF006677) was even more divergent (Figure 3a). A similar pattern of genetic polymorphism was evident in the TPI gene; the sequences from isolates 3460 and 3464 were identical to each other but had six SNPs compared with isolate 3463. This finding suggests that at least two distinct genotypes of *G. microti* were present in muskrats (Figure 3b).

## Discussion

Understanding the taxonomic relationship of a particular group of protozoan parasites that truly reflects biological characteristics and evolutionary relationships is difficult. Most protozoan parasites lack fossil records, are microscopic, and have few informative morphologic and ultrastructural characters; some lack sexual reproduction (*23*,*24*). Although *Giardia* spp. populate the intestinal tracts of almost every group of vertebrates, *G. duodenalis* is the only species found in humans and many other mammals including cattle, cats, dogs, horses, sheep, and pigs (*1*,*25*,*26*). *Giardia* cysts have also been detected in various wild mammals (*14*,*27*–*34*). Although these wild mammals are generally assumed to be infected with *G. duodenalis*, molecular characterization to support this supposition is lacking.

Even though *Giardia* isolates from different mammalian hosts were similar in form, a marked biological diversity among these isolates was noticed in host infectivity (*35*), metabolism (*36*), and in vitro and in vivo growth requirements (*37*,*38*). Multilocus enzyme electrophoresis identified a number of distinct groups of *G. duodenalis* (*39*,*40*). The forgoing heterogeneity suggests that *G. duodenalis* is a species-complex (*39*,*41*,*42*). Phylogenetic characterization based on the nucleotide sequences of GDH, elongation factor 1α (EF1α), TPI, and SSU rRNA genes suggests the presence of five to seven lineages of *G. duodenalis* (*12*,*17*,*43*,*44*). Among the loci analyzed, TPI has the highest degree of polymorphism (*12*). However, only four isolates from humans, two isolates from mice, and one isolate each from cat, dog, pig, rat (*G. muris),* and blue heron (*G. ardeae*) have been characterized at the TPI locus (*12*).

The genetic relationship among various *Giardia* parasites showed by phylogenetic analysis of the TPI gene in this study is largely in agreement with previous observations based on results from the SSU rRNA, TPI, GDH, and EF1α genes (*12*,*17*,*43*,*44*). Thus, on the basis of published and present TPI nucleotide sequences, the following groupings of *G. duodenalis* parasites are evident by all analyses with strong statistical reliability: 1) the formation of a group containing relatively few human isolates (assemblage A); 2) a major group containing most of the human and muskrats isolates, as well as isolates from beavers and a rabbit (assemblage B); 3) the formation of a group containing all isolates from cattle and pigs (assemblage E or the hoofed livestock genotype); 4) the formation of a group containing isolates from dogs (assemblage C); 5) an undefined cat genotype; and 6) an undefined genotype from rats. The assemblage D previously seen in a few dogs (*42*) was not found in this study.

In our study, a distinct and more distant cluster was formed by some isolates from muskrats. DNA sequence analysis of the SSU rRNA gene indicated that these isolates were *G. microti*. This organism was placed between the clades representing the *G. muris* and all the six assemblages of *G. duodenalis*. *Giardia microti* was established as a separate species because of sequence uniqueness of the SSU rRNA gene and minor morphologic differences from *G. duodenalis* (*5*,*6*). Our characterization of TPI nucleotide sequences from muskrats supports the validity of *G. microti*.

Results of phylogenetic analysis are useful in understanding the public health importance of some *G. duodenalis* parasites. Human *G. duodenalis* are placed in two distinct lineages (assemblages A and B), whereas the other four lineages contain only *G. duodenalis* from animals (assemblages C and E, and undefined cat and rat genotypes). One of the assemblages in humans, assemblage B, also contains all beaver isolates and some isolates from muskrats, rabbits, and mice, which strongly suggests that these animal isolates have the potential to infect humans. *Giardia* from beavers has been suggested as the source of infection for backpackers and some waterborne outbreaks of giardiasis (*27*,*30*). Results of our study provide genetic evidence to substantiate these claims.

The TPI-based genotyping tool is also useful in epidemiologic investigations of giardiasis in humans (*15*,*45*,*46*). A recent study in the United Kingdom of sporadic cases of human giardiasis used a TPI-based PCR–restriction fragment length polymorphism genotyping tool. Of the 33 TPI-PCR–positive infected patients, 21 (64%) were infected with assemblage B, 9 (27%) with assemblage A, and 3 (9%) samples were mixed infections of assemblages A and B (*47*). Similar results were obtained with samples from a nursery outbreak, in which 21 (88%) of 24 samples were shown to be *G. duodenlais* assemblage B parasites; the rest were assemblage A parasites (*47*). The intragenotypic variations of TPI in assemblage B identified in the present study should be useful in subtyping outbreak isolates. Because *Giardia* spp. have a clonal population structure (*40*), the use of a typing system based on sequence analysis of a single genetic locus with high sequence heterogeneity, such as TPI, can provide a resolution as high as multilocus sequence typing.

The results of our study suggest that the TPI gene is a good phylogenetic marker for analysis of the molecular evolutionary and taxonomic relationship of *G. duodenalis* parasites. The genetic relationship shown by phylogenetic analysis of the TPI gene is largely in agreement with that obtained at other genetic loci. Results of the molecular analyses support the conclusion that *G. duodenalis* is a species-complex, a finding that should be useful in the revision of *Giardia* taxonomy and standardization of *Giardia* nomenclatures. Results of this study also indicate that *Giardia* parasites from beavers, muskrats, mice, and rabbits represent a potential public health concern.
